# Mapping the potential for offshore aquaculture of salmonids in the Yellow Sea

**DOI:** 10.1007/s42995-022-00141-2

**Published:** 2022-08-18

**Authors:** Shuang-En Yu, Shuang-Lin Dong, Zhi-Xin Zhang, Yu-Yang Zhang, Gianluca Sarà, Jie Wang, Yun-Wei Dong

**Affiliations:** 1 Key Laboratory of Mariculture of Ministry of Education, College of Fisheries, Ocean University of China, Qingdao, 266003 China; 2 Function Laboratory for Marine Fisheries Science and Food Production Processes, Pilot National Laboratory for Marine Science and Technology (Qingdao), Qingdao, 266235 China; 3 CAS Key Laboratory of Tropical Marine Bio-Resources and Ecology, South China Sea Institute of Oceanology, Innovation Academy of South China Sea Ecology and Environmental Engineering, Chinese Academy of Sciences, Guangzhou, 510301 China; 4 Laboratory of Ecology, Department of Earth and Marine Sciences, University of Palermo, 90128 Palermo, Italy

**Keywords:** Aquaculture potential, Offshore aquaculture, *Oncorhynchus mykiss*, *Salmo salar*, Species distribution models, The Yellow Sea Cold Water Mass

## Abstract

Mariculture has been one of the fastest-growing global food production sectors over the past three decades. With the congestion of space and deterioration of the environment in coastal regions, offshore aquaculture has gained increasing attention. Atlantic salmon (*Salmo salar*) and rainbow trout (*Oncorhynchus mykiss*) are two important aquaculture species and contribute to 6.1% of world aquaculture production of finfish. In the present study, we established species distribution models (SDMs) to identify the potential areas for offshore aquaculture of these two cold-water fish species considering the mesoscale spatio-temporal thermal heterogeneity of the Yellow Sea. The values of the area under the curve (AUC) and the true skill statistic (TSS) showed good model performance. The suitability index (SI), which was used in this study to quantitatively assess potential offshore aquaculture sites, was highly dynamic at the surface water layer. However, high SI values occurred throughout the year at deeper water layers. The potential aquaculture areas for *S. salar* and *O. mykiss* in the Yellow Sea were estimated as 52,270 ± 3275 (95% confidence interval, CI) and 146,831 ± 15,023 km^2^, respectively. Our results highlighted the use of SDMs in identifying potential aquaculture areas based on environmental variables. Considering the thermal heterogeneity of the environment, this study suggested that offshore aquaculture for Atlantic salmon and rainbow trout was feasible in the Yellow Sea by adopting new technologies (e.g., sinking cages into deep water) to avoid damage from high temperatures in summer.

**Supplementary Information:**

The online version contains supplementary material available at 10.1007/s42995-022-00141-2.

## Introduction

As of 2020, at least 155 million people across the globe are facing severe hunger, exacerbated by political conflicts, pandemics, and climate change (Food Security Information Network 2021; Laborde et al. 2020; Myers et al. 2017; Puma et al. 2018). To make matters worse, the world’s population is expected to reach 9.7 billion by 2050 (United Nations 2019) and will put enormous pressure on food security. Fisheries, as a key sector of food supplies (Waite et al. 2014), can provide high-quality proteins and nutrients. Since the 1990s, the production of wild capture fisheries has reached a plateau and the production of aquaculture has risen steadily (FAO 2020). Accompanying the rapid development, aquaculture is facing intensive constraints from global change, environmental pollution, and resource conflicts (for example space, fresh water and energy) (Naylor et al. 2021).

Mariculture will be one of the most promising industries to provide food in the future (Costello et al. 2020). However, most mariculture activities have mainly taken place in coastal areas where environmental impacts and resource conflicts with other uses have been accentuated in recent decades (Gentry et al. 2017b; Sarà et al. 2021). To cope with the increasing demand for food, exploring offshore aquaculture has gained increased attention in recent years (Barillé et al. 2020; Gentry et al. 2017b; Lester et al. 2018b; Thomas et al. 2019). Yet, when compared with the rapidly increasing demand for sustainable seafood, the offshore aquaculture industry is still in its infancy (Buck et al. 2017).

Initially, “offshore” aquaculture mainly referred to aquaculture activities located in open waters, some kilometers from the coast, the so-called open-ocean aquaculture (Morro et al. 2021). Recently, the definition of offshore aquaculture has been modified and re-classified according to a variety of criteria, including depth, distance from shore, and wave exposure (Holmer 2010; Lester et al. 2018a; Kapetsky 2013). There is thus inconsistency in definitions of “offshore” aquaculture, and many “offshore” aquaculture activities are relatively closer to shore and in shallower waters than the definition outlined by the Food and Agricultural Organization of the United Nations (FAO) (Froehlich et al. 2017; Lovatelli et al. 2013). Here, we use a broad definition of offshore aquaculture that includes all mariculture that is located in waters that are not directly adjacent to land (Gentry et al. 2017b).

Successfully mapping the potential of offshore aquaculture under changing climatic conditions is essential for the sustainable development of such a sector. The potential regions and yields of some mariculture species have been evaluated on a global scale (Froehlich et al. 2018; Gentry et al. 2017a). The GIS-based Multi-Criteria Evaluation (MCE) combines multiple physical, economic, and socio-ecological factors into structured models, and provides a holistic overview of the potential for a site to successfully support offshore aquaculture (FAO and World Bank 2015; Radiarta et al. 2008). Physical carrying capacity focuses on the effect of physical environmental factors and the farming systems on aquaculture, and is the primary criterion and first stage for aquaculture mapping (Brigolin et al. 2017; Sarà et al. 2018a). Species distribution models (SDMs), both correlative and mechanistic functionally based, use algorithms to evaluate and predict the distribution of species across geographic space and time, and have been widely applied in the fields of conservation planning, resource management, impact evaluation of climate change, and invasive species assessment (Austin et al. 1990; Bosch-Belmar et al. 2022; Elith and Leathwick 2009; Ferrier 2002; Guisan and Thuiller 2005; Peterson 2003; Thomas et al. 2004; Vetaas 2002). An ensemble provides a method for resolving differences among multiple SDMs for the same species (Grenouillet et al. 2011; Pikesley et al. 2013; Scales et al. 2016) and is a reliable tool for assessing the suitability of habitat for the selection of aquaculture sites (Dong et al. 2020). Recently, more studies have suggested that risk assessments at smaller spatial scales helped reveal spatio-temporal heterogeneity that can be ignored in coarse assessments (Payne et al. 2021; Pinsky 2021).

Atlantic salmon (*Salmo salar* Linnaeus, 1758) and rainbow trout (*Oncorhynchus mykiss* Walbaum, 1792) are important aquaculture species, and are widely traded all over the world. The global aquaculture production of *S. salar* and *O. mykiss* in 2018 was 2435.9 and 848.1 kilotonnes, respectively (FAO 2020). However, outdoor farming of salmon and trout in China is facing great challenges due to the lack of appropriate aquaculture areas. In 2018, an offshore platform-based fish farming facility for culturing salmon and trout, Deep Blue 1, was launched in the Yellow Sea Cold Water Mass (YSCWM), 140 nautical miles from the coast (Dong 2019; Huang et al. 2021). With the expansion of aquaculture areas and the deployment of offshore aquaculture facilities, mapping suitable offshore aquaculture areas have been one of the most important priorities for the sustainable development of the industry.

**Fig. 1:**
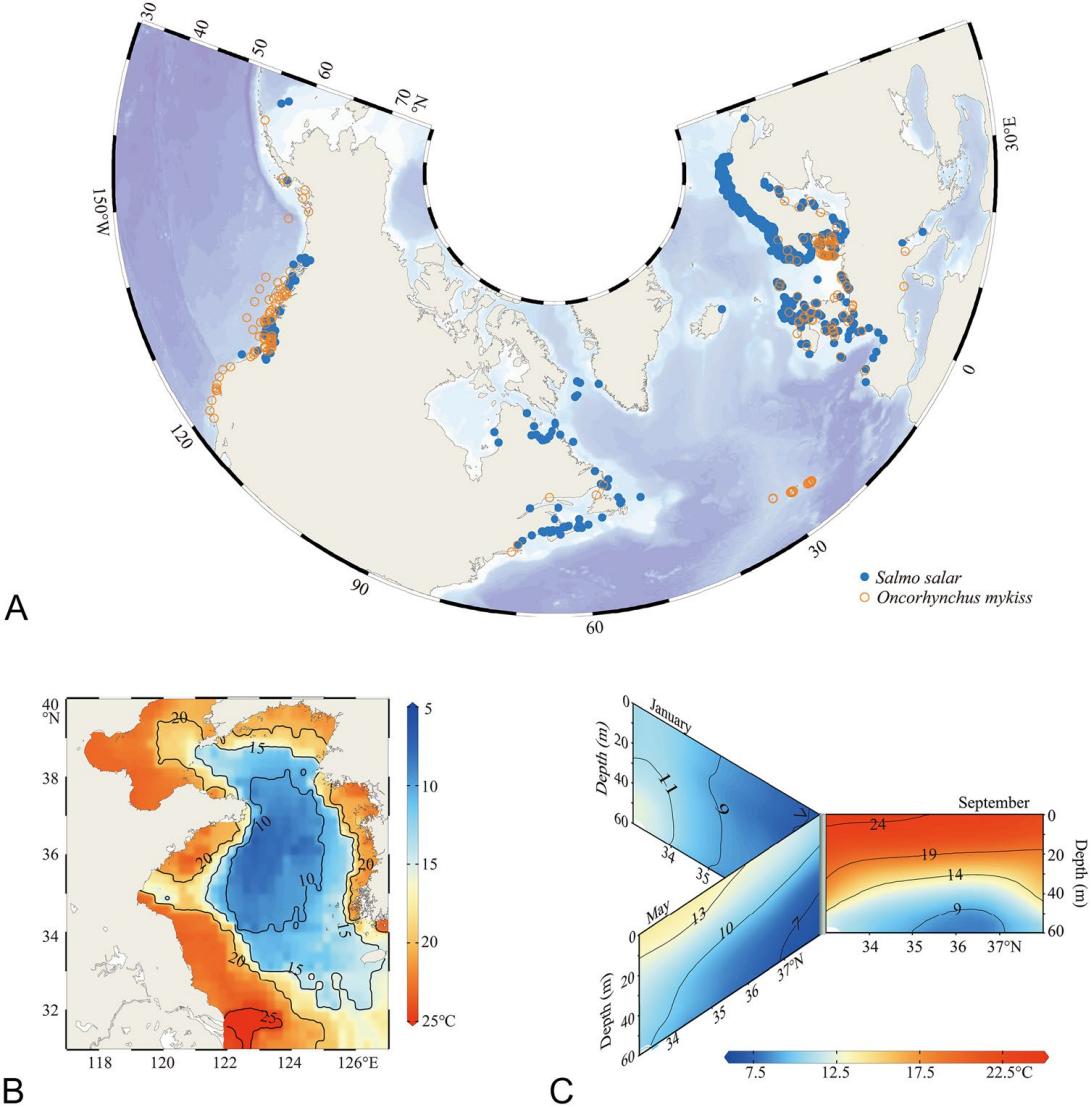
Study areas of the *Salmo salar* and *Oncorhynchus mykiss*. **A** The areas of data collection for developing species distribution models. The blue and orange circles represent the occurrence points for developing species distribution models (SDMs) for *S. salar* and *O. mykiss*, respectively. **B** Location of the Yellow Sea and the sea bottom temperature in September (mean from 1955 to 2017). **C** The vertical section of temperature (mean from 1955 to 2017) in the central Yellow Sea (Longitude: 123.375° E; Latitude: 33°N–38° N). The temperature data of the Yellow Sea were collected from the World Ocean Atlas 2018 dataset

The YSCWM extending from the Liaodong Peninsula to the East China Sea covers one-third of the deep Yellow Sea (Fig. 1B). It forms in May and June, peaks in July and August, and dissipates completely in December, with extremely highly seasonal and vertical variations (Fig. 1C) (Li et al. 2016b; Oh et al. 2013; Park et al. 2011; Yu et al. 2006; Zhang et al. 2008). In summer, the sea surface temperature ranges between 18 and 24 °C, but the temperature of the three cold-water centers (at 50 m or deeper) in the YSCWM is less than 10 °C. With the strong vertical mixing of seawater in autumn, there are large areas of water temperature above 18 °C in the south YSCWM (Li et al. 2016a; Yu et al. 2006), which may affect the aquaculture suitability of the South Yellow Sea. The high dynamic thermal environment in the YSCWM creates an opportunity for farming cold-water fish by providing thermal shelter. In such a heterogeneous thermal environment, it is crucial to explore the fine-scale spatio-temporal patterns of potential aquaculture areas for salmon and trout. For achieving this goal, we developed ensemble SDMs for *S. salar* and *O. mykiss,* and calculated the suitability index (SI) for offshore aquaculture taking into account the spatio-temporal patterns in different water layers. Our study provides useful tools for mapping potential offshore aquaculture areas and emphasizes the importance of considering environmental heterogeneity during the process.

## Results

### Model performances

**Fig. 2:**
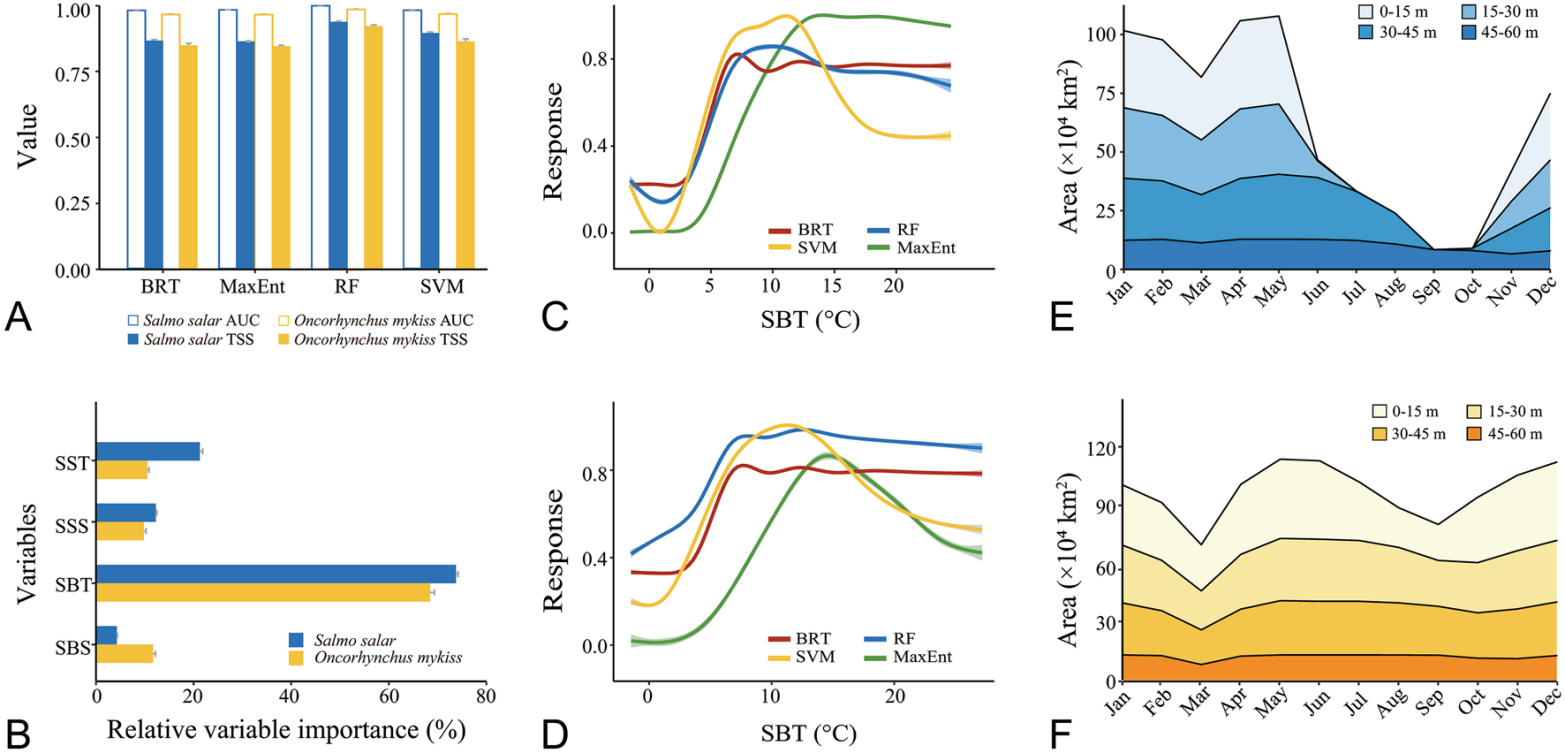
Model performance, variable characteristics, and distribution of areas with high suitability index (SI). **A** The values of the areas under the receiver-operating characteristic curve (AUC) and the true skill statistic (TSS) of the four modeling algorithms for *Salmo salar* (blue) and *Oncorhynchus mykiss* (orange). Results are expressed as mean ± 1 standard error (*N* = 10). **B** Importance of the four variables used to develop the ensemble species distribution models (SDMs) of *S. salar* (blue) and *O. mykiss* (orange). Results are expressed as mean ± 1 standard error (*N* = 10). **C**–**D** The mean response curves of predicted occurrence probability of *S. salar* (**C**) and *O. mykiss* (**D**) against the sea bottom temperature (SBT). Values are mean ± 1 standard error (*N* = 10). **E**–**F** The areas with SI ≥ 0.5 for culturing *S. salar* (**E**) and *O. mykiss* (**F**) in different water layers in the Yellow Sea every month. *RF* random forest, *MaxEnt* maximum entropy model, *SVM* support vector machine, *BRT* boosting regression tree. Four variables include sea surface temperature (SST), sea bottom temperature (SBT), sea surface salinity (SSS), and sea bottom salinity (SBS)

The areas under the receiver-operating characteristic curve (AUC) and the true skill statistic (TSS) were used to assess the accuracy of SDMs. The four modeling algorithms, including random forest (RF), maximum entropy model (MaxEnt), support vector machine (SVM), and boosting regression tree (BRT), had high values of mean AUC (> 0.7) and mean TSS (> 0.4), indicating their good predictive capacities for *S. salar* and *O. mykiss* (Fig. 2A). Among the four modeling algorithms, the RF algorithm had the highest AUC (mean ± standard error, *S. salar*: 0.988 ± 0.001, *O. mykiss*: 0.986 ± 0.002) and TSS values (*S. salar*: 0.928 ± 0.002, *O. mykiss*: 0.923 ± 0.004). For *S. salar*, the BRT and MaxEnt algorithms had the lowest AUC (0.967 ± 0.002) and TSS values (0.852 ± 0.02), respectively. In addition, the MaxEnt algorithm had the lowest AUC (0.965 ± 0.002) and TSS values (0.845 ± 0.004) for *O. mykiss*. Overall, these four modeling algorithms are applicable in constructing the ensemble SDMs for *S. salar* and *O. mykiss*.

### Variable importance and response curves

Among the four variables (sea surface temperature, SST; sea bottom temperature, SBT; sea surface salinity, SSS; and sea bottom salinity, SBS), SBT was the most important environmental variable regulating the potential distribution of *S. salar* (mean relative variable importance ± standard error: 0.736 ± 0.004) and *O. mykiss* (0.683 ± 0.009) (Fig. 2B). Response curves of *S. salar* and *O. mykiss* concerning SBT varied with algorithms (Fig. 2C, D). The mean response curves of *S. salar* and *O. mykiss* suggested that these species had higher probabilities of occurrence in areas with SBT ranging from 5 to 18 °C.

### Suitability index (SI) for offshore aquaculture

**Fig. 3:**
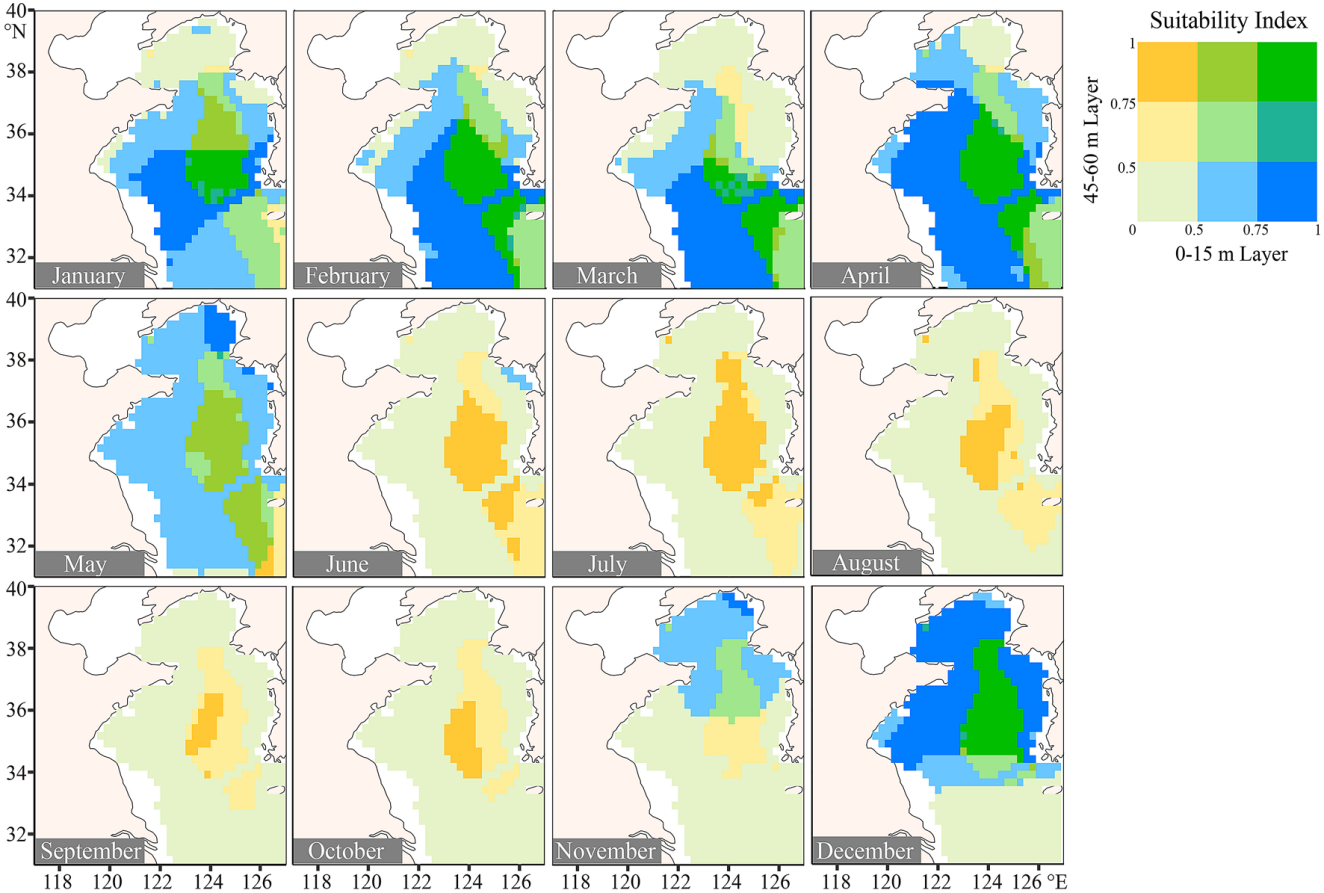
The suitability index (SI) for *Salmo salar* at the layers of 0–15 m and 45–60 m every month in the Yellow Sea. The SI values at 0–15 m water layer and 45–60 m water layer are shown in blue and yellow, respectively. The deepening of green indicates that the values of SI in the two water layers increase isochronously

**Fig. 4:**
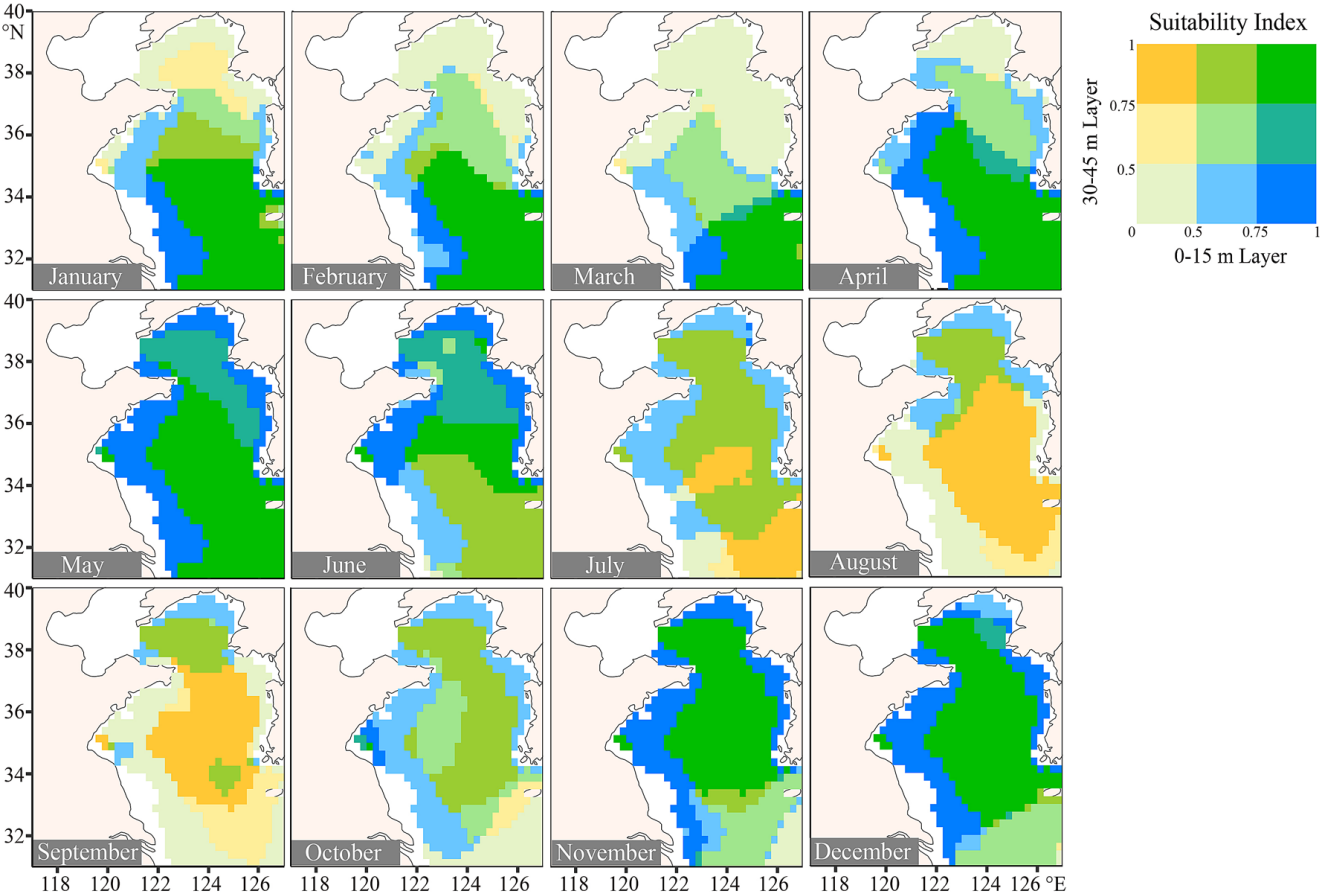
The suitability index (SI) for *Oncorhynchus mykiss* at the layers of 0–15 m and 30–45 m every month in the Yellow Sea. The SI values at 0–15 m water layer and 45–60 m water layer are shown in blue and yellow, respectively. The deepening of green indicates that the values of SI in the two water layers increase isochronously

SI was used to quantitatively assess the offshore aquaculture probability of each grid cell in different months and water layers. The SI values for culturing *S. malar* and *O. mykiss* in the Yellow Sea were highly dynamic in different months and different water layers (Figs. 3, 4).

For *S. salar*, the SI values over most areas of the Yellow Sea were less than 0.5 from June to November at the water layer of 0–15 m. At the water layer of 45–60 m, however, the SI values for culturing *S. salar* remained above 0.5 throughout the year (Fig. 3). The relatively high standard errors of SI values occurred at the 0–15 m layer of the northern Yellow Sea from January to April (Supplementary Figs. S1, S2). These results suggested that farming *S. malar* in the yellow Sea at a depth of 0–45 m was hard to succeed from June to November, but the relatively benign thermal environment at 45-60 m water layer provided a shelter during this period.

The SI values for culturing *O. mykiss* were relatively higher than those for *S. salar*. In most areas of the Yellow Sea, the SI values of *O. mykiss* also decreased in summer (from August to September) at the layer of 0–15 m. At the layer of 30–45 m, however, the SI values remained above 0.5 throughout the year (Fig. 4). The relatively high standard errors of SI values occurred at the 0–15 m layer in the northern Yellow Sea from January to April and at the layer of 30–45 m in March (Supplementary Figs. S3, S4).

### Potential areas for offshore aquaculture

The areas with high SI values (threshold = 0.5) for culturing *S. salar* and *O. mykiss* expanded and contracted seasonally in the Yellow Sea, especially in the surface water layer of 0–15 m (Fig. 2E, F). The areas with high SI values for culturing *S. salar* remained stable at the layer of 45–60 m throughout the year but contracted at the other three shallower layers during the hot seasons. For culturing *O. mykiss*, the areas with high SI values contracted at the layer of 0–15 m from July to September, and the areas in other water layers remained relatively stable. It was worth noting that the areas with high SI values for culturing these two fish were small at the layer of 0-15 m in March (Fig. 2E, F). At this time, the regions with high SI values were mainly located in the southern Yellow Sea (Figs. 3, 4).

We counted the areas of the grid cells for estimating the offshore aquaculture potential based on the SI values (thresholds = 0.4, 0.5, and 0.6) (Table 1). For example, when the SI threshold was set as 0.5, the potential areas for offshore cultivation of *S. salar* and *O. mykiss* were 52,270 ± 3275 (95% confidence intervals, CI) and 146,831 ± 15,023 km^2^, respectively (Table 1).

**Table 1:** Potential areas for offshore aquaculture of *Salmo salar* and *Oncorhynchus mykiss* in the Yellow Sea with different suitability index (SI) thresholds

Species	SI threshold	Mean ± standard error (km^2^)	95% confidence intervals (km^2^)
*Salmo salar*	0.4	64,651 ± 1494	61,692–67,549
	0.5	52,270 ± 1671	48,996–55,545
	0.6	39,608 ± 2033	35,623–43,594
*Oncorhynchus mykiss*	0.4	195,795 ± 3338	189,253–202,337
	0.5	146,831 ± 7665	131,808–161,854
	0.6	62,999 ± 10,079	43,244–82,754

## Discussion

With extensive species occurrences data and environmental data, ensemble species distribution models (SDMs) were constructed for identifying the potential areas of offshore aquaculture for *S. salar* and *O. mykiss*. Our results show that the offshore aquaculture of the salmon and trout in the Yellow Sea is feasible taking into account the mesoscale spatio-temporal environmental heterogeneity. This study provides novel and important information for mapping offshore aquaculture areas in the aspect of physical environment, an essential element to address for aquaculture zoning.

### Considering mesoscale environmental variations in mapping the potential of offshore aquaculture

SDMs can be reliable tools for selecting potential offshore aquaculture areas (Beard et al. 2020; Falconer et al. 2016). Previous studies have confirmed that SDMs were useful for identifying suitable aquaculture sites for farming Manila clam (*Ruditapes philippinarum*) (Dong et al. 2020) and for identifying the suitable locations for seaweeds aquaculture in the Spencer Gulf, South Australia (Wiltshire and Tanner 2020). In the present study, the high values of the areas under the receiver-operating characteristic curve (AUC) and the true skill statistic (TSS) suggest that the SDMs have a good predictive capacity for selecting suitable aquaculture areas for the cold-water fish *S. salar* and *O. mykiss*.

The following factors should consider during the selection of aquaculture areas using SDMs. First, the behavioral response of the aquaculture species is usually restricted to a limited area and then is more constricted than that of the wild species. Second, the offshore aquaculture system is generally a human-manipulated artificial system where the feed and dissolved oxygen usually are not limiting factors with artificial feed supply and aeration. On the other hand, some environmental factors which are difficult to regulate in the open ocean, such as temperature and salinity, are crucial variables for the success of aquaculture. Therefore, these uncontrollable factors should be considered. Third, SDMs for offshore aquaculture should be designed at a high resolution because most aquaculture activities were carried out in relatively small local areas, which can vary in their environmental conditions. The spatio-temporal environmental heterogeneity in the specific aquaculture areas is usually ignored in coarse assessments (Payne et al. 2021; Pinsky 2021; Sarà et al. 2018b). Finally, this study shows the potential value of farming operations that permit growing at variable depths, to ensure that fish remain within optimal temperatures.

### Feasibility of offshore aquaculture of salmonids in the temperate region

Considering the spatio-temporal environmental heterogeneity, offshore aquaculture of salmon and trout is feasible in the Yellow Sea. In the present study, the suitability index (SI) was applied as an indicator to evaluate the suitability of offshore aquaculture for *S. salar* and *O. mykiss,* and quantitatively evaluate the potential farming areas. As indicated by the SI values, the deeper water layers in the Yellow Sea during the hot season provide ‘cool refugia’ for farming salmon and trout in the temperate regions. Sizes of the area with high SI values are highly variable in different water layers and months, and so closely tracking the dynamics of areas with high SI values can increase the feasibility of offshore aquaculture for salmon and trout in the Yellow Sea. In addition, the development of aquaculture facilities (e.g., Deep Blue 1) and the high demands for salmon and trout in the Chinese market are driving forces for the offshore aquaculture of salmonids in the Yellow Sea (Fishery Bureau of Ministry of Agriculture People’s Republic of China 2020; Shi et al. 2021).

### Recommendations for offshore aquaculture of salmonids

The results of this study and other related work provide multiple recommendations for offshore aquaculture in the Yellow Sea.

1. Sinking the net cages into the specific water layer during the hot seasons: restricted by policies, economic costs, and technologies, offshore aquaculture facilities are difficult to move around freely. Based on the results from the present study, vertical adjustments of the positions of the cages can avoid the thermal stress in summer in the Yellow Sea. It suggests that the cages can be sunk into the water layer of 45–60 m from June to November for culturing *S. salar*, and into the water layer of 30–45 m from August to September for culturing *O. mykiss*.
2. Long-term environmental monitoring in the mariculture areas: environmental variations have huge impacts on mariculture, and so it is crucial to conduct in situ real-time monitoring of the aquaculture environmental factors, such as temperature (Elliott and Elliott 2010), dissolved oxygen (Oldham et al. 2019) or currents (Castro et al. 2011; Nilsen et al. 2019), for evaluating and predicting the effect of environmental factors on aquaculture and for making decisions to avoid or reduce losses in the face of environmental stresses.
3. Assessing and controlling environmental impacts of offshore aquaculture: the impacts of mariculture on the surrounding environment are complex and diverse (Gentry et al. 2017b). Uneaten feed, feces, chemicals, antibiotics, and even dead fish can pollute the environment (Cao et al. 2007; Navedo and Vargas-Chacoff 2021; Price and Morris 2013; Reverter et al. 2020; Seymour and Bergheim 1991). For example, in March 2021, the mass death of farmed salmon threatened the coastal environment in Patagonia, South America (Navedo and Vargas-Chacoff 2021). Integrated multitrophic aquaculture (IMTA) may be an effective approach to mitigate pollution from aquaculture by imitating natural ecological nutrient cycling (Troell et al. 2009) overall increasing the resilience of the aquaculture systems as testified by farmers around the world under the COVID pandemic (Sarà et al. 2021). Studies such as these exploring the optimum growth conditions should be accompanied by studies to evaluate sustainability based on potential environmental damage (Sarà et al. 2018a).
4. Preventing genetic risks from farmed fish escapes: large-scale escapes of farmed fish can cause serious genetic risks. From 2001 to 2009, 3.93 million *S. salar*, 0.98 million *O. mykiss*, and 1.05 million *Gadus morhua* had escaped in Norway (Jensen et al. 2010) and large-scale salmon escape also occurred in Chile in 2018 (Gomez-Uchida et al. 2018). Escapes of fish from aquaculture systems can affect the structure of the local food webs and gene pools (Weir and Grant 2005), and transfer aquaculture-associated diseases (Arechavala-Lopez et al. 2018; Bouwmeester et al. 2021; Jensen et al. 2010). Mandatory management, scientific assessment, high technical standards for mariculture facilities, and rigorous operation can effectively reduce the impacts of escape events (Jensen et al. 2010).
5. Ensuring the health of farmed fishes: sea lice and amoebic gill disease (AGD) are common diseases in the aquaculture of salmon and trout. These diseases have serious effects on finfish health and cause economic losses (Carvalho et al. 2020; Shinn et al. 2015). Although the probability of sea lice infecting fish from their origin farm is low in offshore aquaculture sites (Kragesteen et al. 2018), the consequences of this disease should be serious due to the high density of farmed fish (Morro et al. 2021). Cutting the spread of diseases and conducting real-time health monitoring of farmed fish are essential to ensure the health and welfare of fish.

### Limitations and perspectives

1. Climate change: aquaculture has encountered dramatic changes in temperature, pH, dissolved oxygen, sea level, and extreme events in the face of climate change (Barange and Perry 2009; Handisyde et al. 2006; Ma et al. 2021). Climate change increases the complexity and uncertainty of aquaculture systems (Froehlich et al. 2018; Gentry et al. 2017a; Handisyde et al. 2017; Klinger et al. 2017). IPCC’s Sixth Assessment Report (AR6) has provided more reliable future environmental projections on the regional scale (Doblas-Reyes et al. 2021). Using these regional scenario projections of future climate, SDMs can be applied to assess the vulnerability of offshore aquaculture in the future for the sustainable development of offshore aquaculture.
2. Mechanistic models: incorporating the mechanistic relationships between the functional traits of organisms and their environments into SDMs for aquaculture can provide more useful information (e.g., Bosch-Belmar et al. 2022; Liao et al. 2021). Thermal performance curves (TPCs) have been used to predict the potential productivity for three common aquaculture species (*S. salar*, *Sparus aurata*, and *Rachycentron canadum*), representing different thermal guilds across species and regions, in the context of global warming (Klinger et al. 2017). The species’ temperature tolerance range (maximum and minimum temperature) and von Bertalanffy growth function (VBGF) parameters (K and Linf) have been used to assess the relative potential productivity across countries (Gentry et al. 2017a). Based on the energy budget of an individual organism throughout its life cycle, the Dynamic Energy Budget (DEB) model can provide quantitative information for aquaculture, and has been used widely to estimate the potential for aquaculture species (Bertolini et al. 2021; Sarà et al. 2012, 2018a).
3. Carrying capability: ecosystem Approach to Aquaculture (EAA) emphasizes the integration of aquaculture activities within the wider ecosystem (Corner and Aguilar-Manjarrez 2017). Our study focused on the effect of the physical environment on the mapping of aquaculture areas, while the Multi-Criteria Evaluation (MCE) also needs to fully consider the ecological carrying capability, production carrying capability, and social carrying capability in the process of actual aquaculture spatial planning (Filgueira et al. 2015). The production carrying capability can assess the maximum level of aquaculture production from a biomass or economics perspective. The assessment of ecological carrying capability considers the whole ecosystem involved in aquaculture (McKindsey et al. 2006), and the assessment of social carrying capacity limits the amount of aquaculture by mitigating potential conflicts across different uses of marine space or resources.
4. Socio-economic factors: besides the physical environment, socio-economic factors also should be considered on this physical “base map”. For example, social opposition and complex and uncertain regulatory and permitting policies have hampered the development of aquaculture in the United States (Knapp and Rubino 2016; Lester et al. 2018a). Government regulations limited the commercial development of offshore aquaculture, particularly in the USA and European Union, due to disputes over its interactions with the environment, ecological damage, and conflicting uses of space resources (Gentry et al. 2017b; Ramos et al. 2017). Offshore aquaculture activities require large-scale farming systems (Naylor et al. 2021) and rely on high technology and costs for development, construction, and maintenance. Meanwhile, other marine management also need to be fully considered in the implementation of aquaculture projects, such as shipping activities, marine conservation areas, and fishing (Gentry et al. 2017b). For example, spatial conflicts between shipping and aquaculture as well as the potential for long-distance transport of pathogens are often considered in aquaculture spatial planning (Gimpel et al. 2018; Murray et al. 2002). Besides, co-location of aquaculture and other uses (e.g., offshore wind farms) could be an effective way to mitigate conflicts (Stelzenmüller et al. 2016). As the interactions between offshore aquaculture and other marine spatial management may be synergistic or conflicting (Lester et al. 2013), a holistic solution should be exploited for the sustainable development of offshore aquaculture in regions with suitable physical environments.
5. Prerequisites for using aquaculture SDMs: moving to a new environment may lead to changes in a species’ niche from its original niche (Broennimann et al. 2007; Ørsted and Ørsted 2019; Torres et al. 2018), and cause transferability issue of SDMs. Previous studies have highlighted the usefulness of SDMs to predict species invasions (Peterson 2003; Peterson and Vieglais 2001; Ørsted and Ørsted 2019; Torres et al. 2018) based on “Niche conservatism” (Petitpierre et al. 2012). In the present study, we assumed that there were no niche shifts for *S. salar* and *O. mykiss* after being moved to the Yellow Sea. However, model transferability deserves more research attention.

## Conclusion

To map the potential for offshore aquaculture of the salmonids in the Yellow Sea, we conducted ensemble species distribution models (SDMs) for Atlantic salmon (*S. salar*) and rainbow trout (*O. mykiss*) and assessed the suitability index (SI) for culturing these two cold-water fish in the Yellow Sea. Our results enabled us to estimate the potential areas for culturing *S. salar* and *O. mykiss*. Overall, the sizes of the area with high SI values for farming *S. salar* and *O. mykiss* are highly variable in different water layers and different months. The offshore aquaculture for salmonids should be feasible in the Yellow Sea by sinking cages into deep water to avoid damage from high temperatures. Therefore, SDMs are useful tools for estimating physical capability in aquaculture zoning. For the future expansion of offshore aquaculture, mesoscale spatio-temporal environmental heterogeneity needs to be fully considered. Notably, this study points to a potential optimum based on theoretical ecological suitability. The assumptions of niche conservation, local complex socio-economic factors and other marine spatial planning should be considered in offshore aquaculture zoning.

## Materials and methods

### Species occurrences data

The global distribution data of *S. salar* and *O. mykiss* was downloaded from the Global Biodiversity Information Facility (GBIF, https://www.gbif.org/) (GBIF 2021a, b), and 518,234 occurrences of *S. salar* and 202,792 occurrences of *O. mykiss* were acquired (Fig. 1A). The dataset provides a large dataset of occurrences data records around the world, covering the main distribution areas of *S. salar* and *O. mykiss*. To identify the potential areas for offshore aquaculture, only the marine occurrences data (terrestrial and freshwater occurrences data were eliminated) of target species with coordinates were retained, and the duplicate and wrong data were eliminated. To reduce the effects of sampling bias, and retain the greatest amount of useful information, the R package *spThin* was used to return a dataset with the maximum number of records for a thinning distance of 10 km (close to 5 arcmin resolution grids) with 100 iterations (Aiello-Lammens et al. 2015). Overall, 623 records and 181 records were used to develop species-specific species distribution models (SDMs) for *S. salar* and *O. mykiss*, respectively.

### Environmental data

Dissolved oxygen, food, hydrodynamic characteristics, water temperature, and salinity are important variables for fish distribution patterns of natural populations (Pickens et al. 2021). It was evaluated whether these variables should be involved in the modeling. In the Yellow Sea Cold Water Mass (YSCWM), dissolved oxygen (DO) in most areas is above 6 mg/L throughout the year (Xin et al. 2013), which meets the physiological demands of dissolved oxygen for the cultured fish, and also aeration can be supplied in case of hypoxia in offshore aquaculture (Dong 2019). The high DO and artificial oxygen supply guarantee the requirements of farmed fish for oxygen. During the culturing period, fish usually were fed to excess with formulated diets, so food usually should not be a limiting factor. Farmed fish was limited in a net cage, and so hydrodynamic characteristics, e.g., wave and current velocity, were not involved in modeling although these factors can affect the distribution in the field. Seawater temperature is one of the biggest challenges for limiting the distribution of salmon and trout (Elliott and Elliott 2010; Mishra et al. 2021), and also for farming salmonids in the Yellow Sea. Salinity represents a critical environmental factor for the farmed fish and cannot be manipulated in the ocean, so it is potentially important for offshore aquaculture. Thus, in the present study, environmental factors including sea surface temperature (SST), sea bottom temperature (SBT), sea surface salinity (SSS), and sea bottom salinity (SBS) were selected as variables used to develop the ensemble species distribution models for mapping the potential of offshore aquaculture.

The R package *sdmpredictors* (Bosch and Fernandez 2021) were used to download the environmental data (from 2000 to 2014) represented on a latitude–longitude grid at 5 arcmin resolution from Bio-ORACLE v2.1 (https://www.bio-oracle.org/) for developing models (Assis et al. 2018). Dataset names included Sea water temperature (mean at mean depth), Sea surface temperature (mean), Seawater salinity (mean at mean depth), and Sea surface salinity (mean). To check the collinearity between environmental variables, the variance inflation factor (VIF) of the four environmental variables was calculated for *S. salar* and *O. mykiss*, respectively. All variables met the criteria (VIF ≤ 10) and were selected for modeling (Belsley et al. 1980).

For calculating the suitability index (SI) in different water layers in the Yellow Sea, we downloaded the monthly averaged global environmental data (from 2005 to 2017) in the NetCDF format from the World Ocean Atlas 2018 (https://www.ncei.noaa.gov/access/world-ocean-atlas-2018/), with a spatial resolution of 15 arcmins (Locarnini et al. 2018; Zweng et al. 2018), in different water depths at a 15 m interval, 0–15 m, 15–30 m, 15–45 m, and 45–60 m. Environmental data of the Yellow Sea were cropped from this global dataset to avoid the potential bias due to the scale effect between the global and regional climate models on SDMs.

All the environmental data were analyzed using R version 4.0.3 (R development Core Team 2020) with the following packages: *ncdf4* (Pierce 2019), *sp* (Pebesma and Bivand 2022), and *raster* (Hijmans 2020).

### Modeling and suitability index assessment

Previous studies have shown that the species’ niche may change due to the adaptation of species to a new environment, the behavior of aquaculture species, and the density-dependent effect when moving to new environments (Ørsted and Ørsted 2019; Torres et al. 2018), which may cause transferability issues of SDMs. In the present study, we aimed to identify suitable physical environments for *S. salar* and *O. mykiss* in the Yellow Sea using the correlative SDMs and developed SDMs under the assumption that the niches were conservative (Ackerly 2003).

We developed SDMs with four algorithms, including random forest (RF) (Breiman 2001), maximum entropy model (MaxEnt) (Phillips et al. 2006), support vector machine (SVM) (Cortes and Vapnik 1995), and boosting regression tree (BRT) (Elith et al. 2008) using R package *sdm* (Naimi and Araújo 2016). These algorithms have been widely used in SDM construction and application (Pendleton et al. 2020; Pouteau et al. 2011; Valavi et al. 2021). As the distribution information was presence-only data, using the argument “bg”, we randomly simulated pseudo-absence points for RF, SVM, and BRT in a 1:1 ratio, and also simulated 10,000 pseudo-absence points for MaxEnt (Phillips et al. 2009; Sillero and Barbosa 2021). In total, we generated 10 different pseudo-absence data sets and repeated the complete modeling and prediction process. We applied 75% of the dataset for training the models and the remaining 25% for testing using bootstrapping to generate 10 replicates for each algorithm. Meanwhile, the environmental variables were set to the “predictor” parameter.

The areas under the receiver-operating characteristic curve (AUC) and the true skill statistic (TSS) were used to assess the accuracy of SDMs (Allouche et al. 2006; Swets 1988). The AUC provides an indication of the usefulness of the models for prioritizing areas in terms of their relative importance as habitats for a particular species (Hanley and McNeil 1982). The TSS is presented as an improved measure of model accuracy, defining the average of the net prediction success rates for presence sites and absence sites (Allouche et al. 2006). The SDMs with TSS > 0.40 and AUC > 0.70 were selected for developing the weighted average ensemble models of *S*. *salar* and *O*. *mykiss* (Araújo et al. 2005; Engler et al. 2011). We used the function “getVarImp” to assess the contribution of each predictor variable to model fit, and the function “rcurve” to get the relationship between the probability of occurrence and each of the predictor variables.

The ensemble models of all four algorithms for each month and water layer were conducted by the function “ensemble”. The TSS values which have been shown to work better in previous studies were specified as a weighting factor for creating the ensemble SDMs (Alabia et al. 2016) and the environment variables were set to weight factor and predictors, respectively.

The suitability index (SI) values (between 0 and 1) were used to quantitatively assess the suitability of offshore aquaculture and estimated the potential areas for offshore aquaculture as the threshold. The SI value of each algorithm was calculated by the function “predict”, and the weighted average of SI was calculated based on the TSS values (Alabia et al. 2016, 2020; Mugo and Saitoh 2020). The higher value of SI represents a higher potential in the farming areas.

A grid was considered to have offshore aquaculture potential if a farmed species acquired habitats with SI values above the threshold only by adjusting the depth of the cage vertically throughout the year. Distinguished from previous SDMs studies, which often used a 10th percentile threshold to transform continuous habitat suitability into binary maps (Pearson et al. 2007), three different SI thresholds (0.4, 0.5, and 0.6) were used in the present study. The grid cells with SI values high the thresholds were regarded as suitable areas for offshore aquaculture. The area of each grid cell was ~ 623.75 km^2^ estimated by the actual area of the grid at 36°N.

All the statistical analyses on the results were performed in R version 4.0.3 (R development Core Team, 2020).

## Supplementary Information

Below is the link to the electronic supplementary material.

Supplementary file1 (DOCX 1520 KB)

## Data Availability

The data underlying this article are available in the article and online supplementary materials.
