# Supplementary material for: Mapping the potential for offshore aquaculture of salmonids in the Yellow Sea
Source: Mar Life Sci Technol. 2022 Aug 18;4(3):329–42. doi: 10.1007/s42995-022-00141-2 (PMC10077287; doi:10.1007/s42995-022-00141-2)
Supplement: Supplementary file 1 — Supplementary file1 (DOCX 1520 KB) [file 42995_2022_141_MOESM1_ESM.docx]

**Supplementary materials for**

**Mapping the potential** **for offshore aquaculture of** **Salmonids in the Yellow Sea**

Shuang-En Yu^1^, Shuang-Lin Dong^1,2^, Zhi-Xin Zhang^3^, Yu-Yang Zhang^1^, Gianluca Sarà^4^, Jie Wang^1*^, Yun-Wei Dong^1,2^^*^

1. Key Laboratory of Mariculture of Ministry of Education, College of Fisheries, Ocean University of China, Qingdao 266003, China
2. Function Laboratory for Marine Fisheries Science and Food Production Processes, Pilot National Laboratory for Marine Science and Technology (Qingdao), Qingdao 266235, China
3. CAS Key Laboratory of Tropical Marine Bio-resources and Ecology, South China Sea Institute of Oceanology, Innovation Academy of South China Sea Ecology and Environmental Engineering, Chinese Academy of Sciences, Guangzhou 510301, China
4. Laboratory of Ecology, Department of Earth and Marine Sciences, University of Palermo, Palermo 90128, Italy

^*^Corresponding author: [dongyw@ouc.edu.cn](mailto:dongyw@ouc.edu.cn), wangjie@ouc.edu.cn


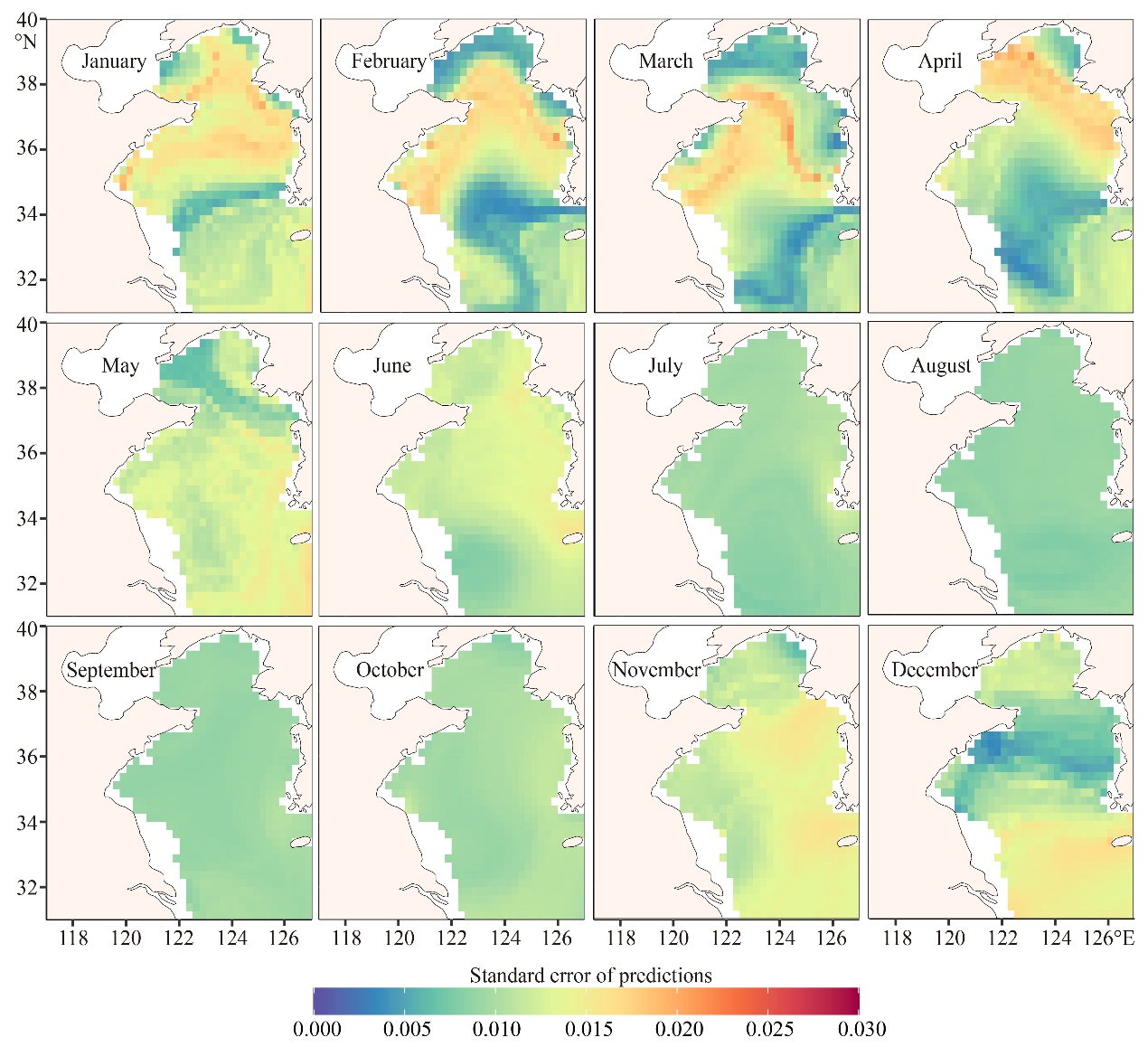


**Supplementary Fig. S1** Standard errors of suitability index (SI) values for culturing *Salmo salar* at the layer of 0-15 m.


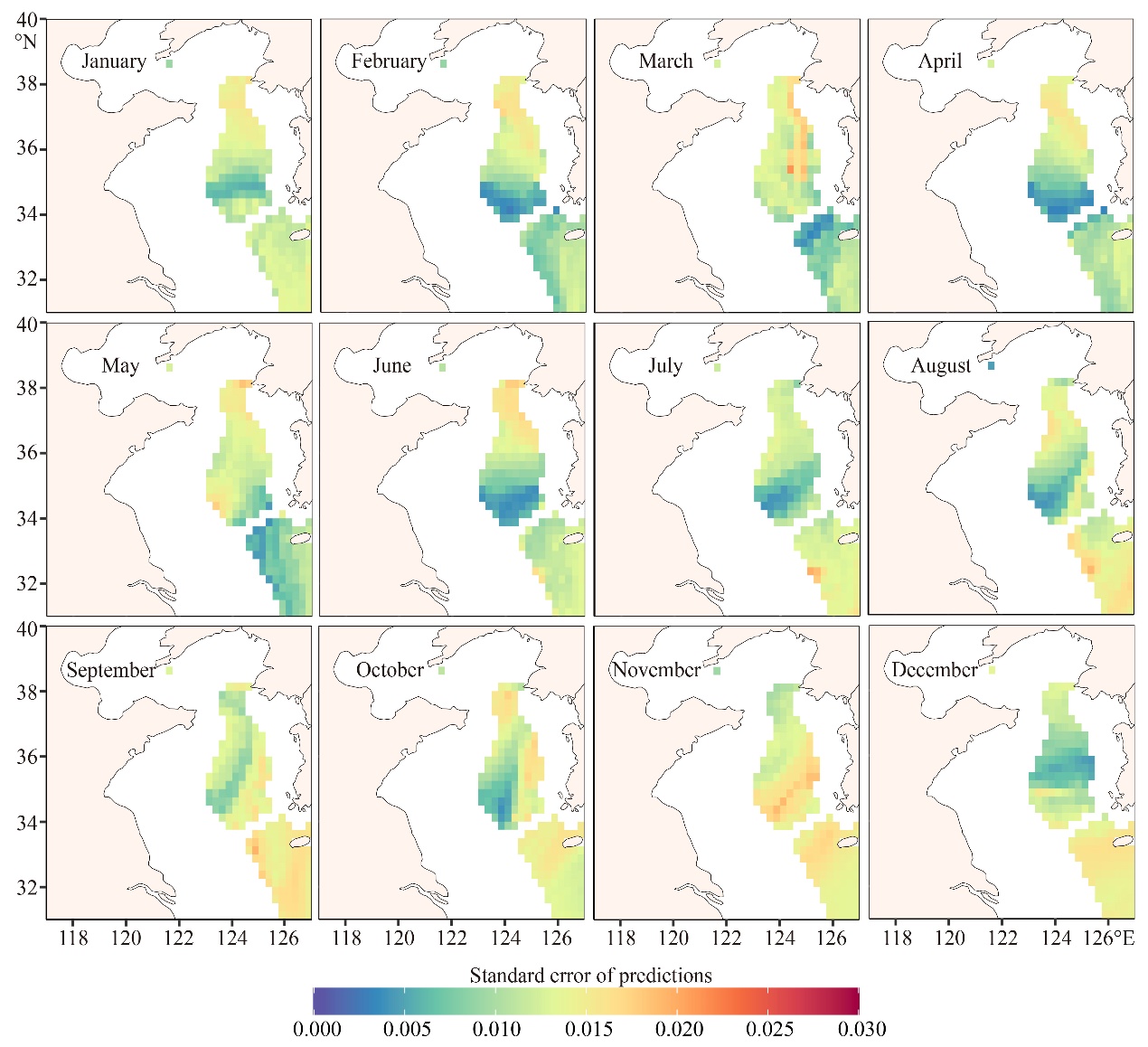


**Supplementary Fig. S2** Standard errors of suitability index (SI) values for culturing *Salmo salar* at the layer of 45-60 m.


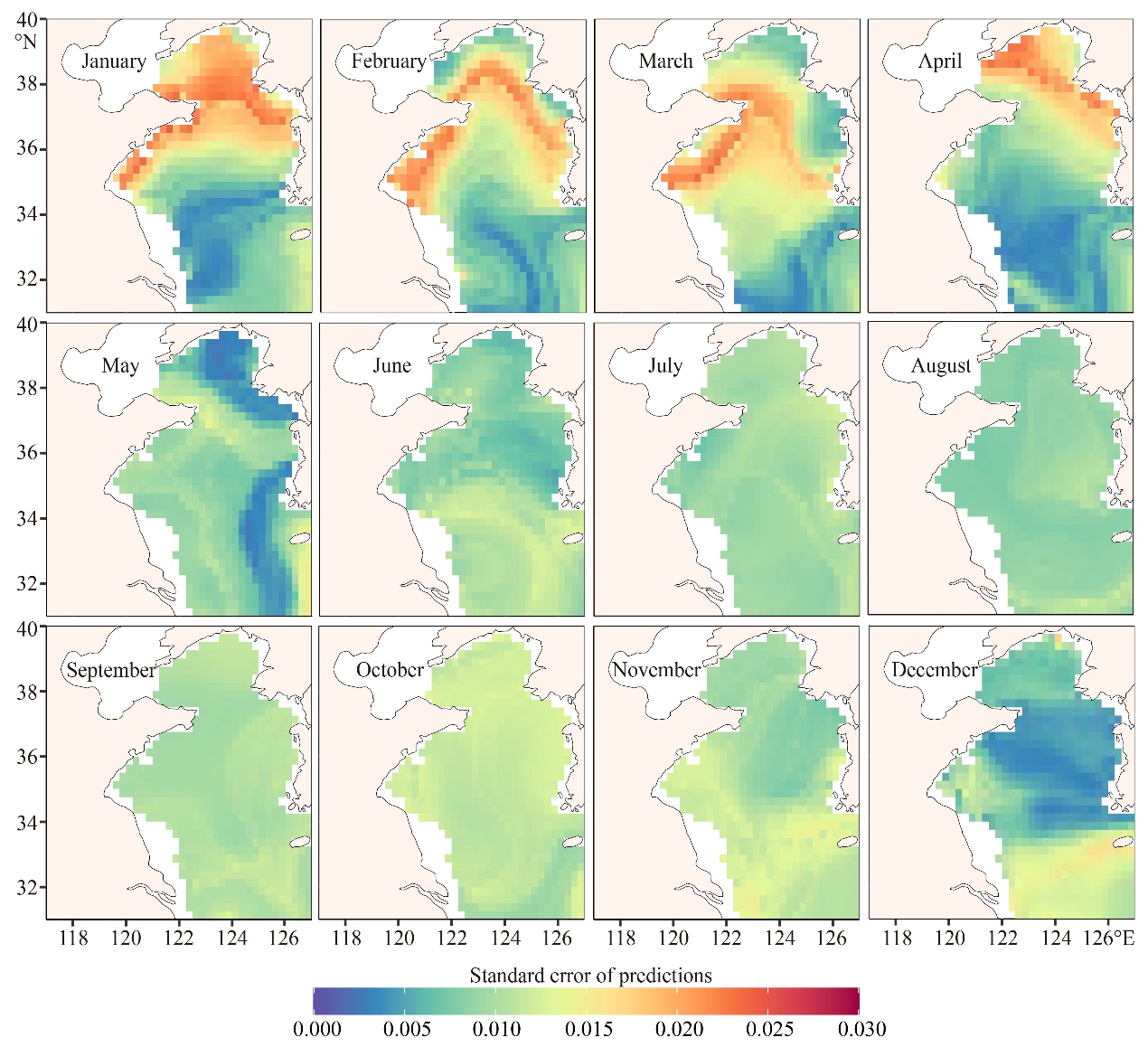


**Supplementary Fig. S3** Standard errors of suitability index (SI) values for culturing *Oncorhynchus mykiss* at the layer of 0-15 m.


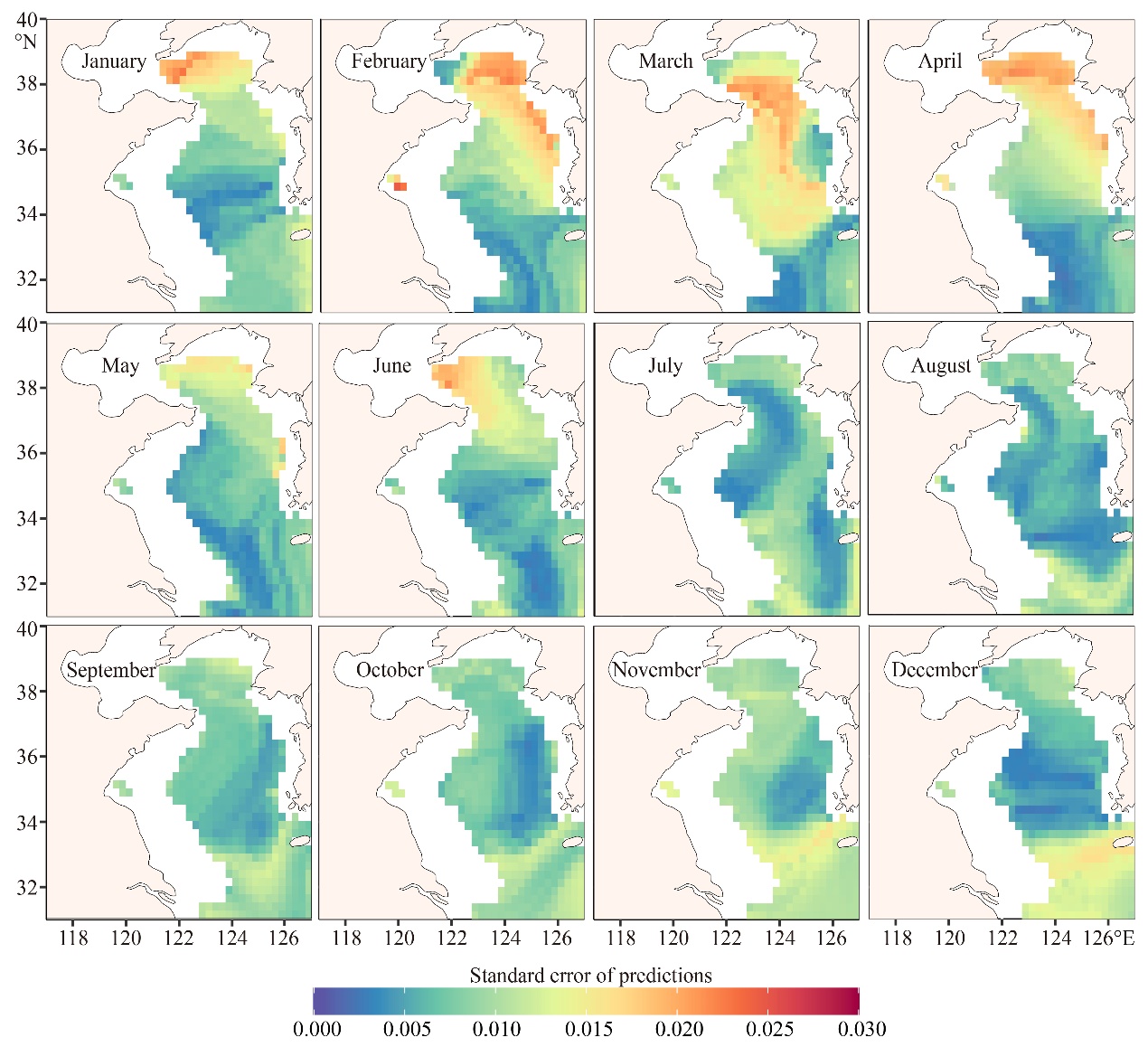


**Supplementary Fig. S4** Standard errors of suitability index (SI) values for culturing *Oncorhynchus mykiss* at the layer of 30-45 m.
